# Microblog credibility indicators regarding misinformation of genetically modified food on Weibo

**DOI:** 10.1371/journal.pone.0252392

**Published:** 2021-06-01

**Authors:** Jiaojiao Ji, Naipeng Chao, Shitong Wei, George A. Barnett

**Affiliations:** 1 Department of Science and Technology Communication, University of Science and Technology of China, Hefei, Anhui, China; 2 School of Communication, Shenzhen University, Shenzhen, Guangdong, China; 3 Department of Statistics, University of California, Davis, CA, United States of America; 4 Department of Communication, University of California, Davis, CA, United States of America; Fuzhou University, CHINA

## Abstract

The considerable amount of misinformation on social media regarding genetically modified (GM) food will not only hinder public understanding but also mislead the public to make unreasoned decisions. This study discovered a new mechanism of misinformation diffusion in the case of GM food and applied a framework of supervised machine learning to identify effective credibility indicators for the misinformation prediction of GM food. Main indicators are proposed, including user identities involved in spreading information, linguistic styles, and propagation dynamics. Results show that linguistic styles, including sentiment and topics, have the dominant predictive power. In addition, among the user identities, engagement, and extroversion are effective predictors, while reputation has almost no predictive power in this study. Finally, we provide strategies that readers should be aware of when assessing the credibility of online posts and suggest improvements that Weibo can use to avoid rumormongering and enhance the science communication of GM food.

## Introduction

The issue of genetically modified food has become a topic of concern for the Chinese people. Currently, misinformation about GM food is spreading rapidly across the most popular microblog in China, i.e., Sina Weibo. For instance, a widespread falsehood is that GM food will cause cancer, infertility, and autism. Another popular one is that Americans do not eat GM food, while the truth is that a lot of processed foods containing ingredients from engineered canola, soybeans, and corn have been sold in the supermarkets for a long time [1]. A recent report indicates that most online users are negative, and the debate on this issue is almost one-sided [2]. Negativity and resistance to GM food could be explained by the imagination about genetic modification, the perceived absence of benefits, and food and culture [3]. It is very difficult to reduce this kind of resistance or opposition even with scientific evidence [4]. What’s worse, the unfamiliarity with genetically modified foods may contribute to the presence of misinformation. The majority of people have limited knowledge about GM technology, and only a small proportion can understand the related scientific principles [5]. In the absence of relevant knowledge, people are susceptible to misinformation [6]. They would not be able to debunk misinformation or be misinformed easily, which will increase GM food misinformation. Generally, being misinformed could be interpreted as holding inaccurate views and being uninformed about scientific facts and processes [7]. Misinformation may manipulate social media discourse about GM technology and impede appropriate public discussion. Therefore, misinformation is likely to influence the policy-making process and perhaps hinder the adoption of GM technology.

Though some risks of misinformation about GM food are well known, and the mitigating measures have also been studied, the trickiest challenge lies in identifying GM food’s misinformation from the massive user-generated content on social media Weibo. Social media platforms have changed the manner people seek information about societal issues and the way they find out about the latest science stories [8]. People have stepped into the era of big data and have been immersed in the explosively grew data of social media platforms. However, these platforms usually fail to halt the spread of misinformation and make users exposed to potential risks [9] due to the explosion of user-generated content and the inadequacy of fact-checking efforts. Misinformation spreads very quickly on social media [10], and fact-checking information lags behind the misinformation by 10–20 hours [11]. Particularly, 45% of online rumors concern food safety, and rumors about GM food are widespread in China [5].

To cope with this challenge, the computational social sciences’ actions and methods are needed to improve the fact-checking efficacy. A growing number of social scientists are leveraging the capacity to collect and analyze data at an unprecedented scale [12] and taking advantage of computational methods, including machine learning, network analysis, natural language processing, and other statistical tools for the measurement of unstructured social data [13]. Also, a growing body of literature has independently addressed various indicators of rumors broadly. However, few studies have specifically examined false rumors of food science, especially GM food.

The current study aims to understand the mechanism of GM food misinformation diffusion and propose a comprehensive and practical example of the framework of supervised machine learning to identify effective indicators to predict the misinformation of GM food on Weibo. We will discuss strategies that users should be aware of when assessing the credibility of the posts and suggest design improvements that platforms can use to display searching results better so as to enhance science communication. Practically speaking, exploring credibility indicators will help people identify misinformation efficiently, enhance a scientific understanding of GM food, and provoke appropriate public discussion, which will contribute substantially to the policy-making process. The paper is organized as follows. In the next section, we propose indicators based on the existing literature. We then build up a reliable domain-specific dataset for further training and prediction. After that, we train the classifiers and evaluate the performance of the three sets of indicators and all the individual indicators.

### Credibility indicators of misinformation

Misinformation usually refers to false or inaccurate information, which may unintentionally deceive or mislead the information receiver, while disinformation is deliberately deceptive information [14–16]. Rumor often refers to unverified information that can be either true or false. For clarity, in this study, misinformation refers to false rumors irrespective of whether it is deliberate or accidental and has already been falsified by credible sources, such as the government, the scientific community, news media, and journals [17]. For example, some users may spread “people in the United States do not eat GM food” deliberately or accidentally, which will be defined as misinformation in this study. The terms “misinformation” and “false rumor” will be used interchangeably hereafter. Moreover, the identification of misinformation often starts with the study of credibility indicators, which are mainly developed from two streams: one is the classification-based approach that uses automatic methods like machine learning techniques for assessing the credibility of tweets [18, 19]; the other one is the survey-based approach that looks into factors affecting perceived credibility of posts based on survey or experiments [20, 21]. For both streams, the broader types of misinformation could be detected from three categories of information [19, 21, 22]: identities of the users involved in spreading information, linguistic styles used to express rumors, and propagation dynamics, which are widely used in credibility assessment research. This study will investigate the three categories of features as presented by Weibo and available directly to readers.

In rumor studies on microblogs, scholarly attention of user identities mainly centered on users’ profiles [9], especially their engagement and reputation. Engagement refers to the users’ seniority and activity level on social media since joining, which is deemed to relate to the credibility of users and posts. The longer a user has been using microblog, the more likely he has developed significant connections with others, and the more likely he would be viewed as a credible user. And more active users tend to spread more credible information, and these credible users propagate trustworthy news [18]. And reputation builds on the user’s connection relationships on microblogs and is one of the prevalent heuristics used to evaluate credibility [23]. It has been found the reputation has something to do with the rumormongering behavior, of which the mechanism, however, is still not fully understood [2, 24, 25]. Also, some took the personality and gender of the users into account. Users being extraverted on social media would spread misinformation to interact with others for socializing purposes, as spreading rumors can be regarded as a way of enhancing relationships, including building and maintaining relationships [26, 27]. As for gender, there is no unanimous conclusion about gender differences in spreading rumors [27–30]. Therefore, both extroversion and gender are included as indicators for misinformation prediction of GM food.

Linguistic styles are also known as message-based features, which examine characteristics of the posts, such as topics, length, syntax, and sentiment score, among which sentiment and topic are extensively used and effective [31]. Also, non-specific authority reference in posts can be used as a predictor of misinformation [32]. The relationship between the sentiment and credibility has been intensively studied on different topics [18, 33–36]. Some found that false rumors often appear along with the negative sentiment, while the other shows that tweets with negative sentiments tend to be credible information. In addition, the relationship between rumor strength and topic importance of the subject to the individual concerned was discussed decades ago [37]. And employing text analytics to study topics for detecting deceptive information has been proven effective [38–40]. In this study, we infer that misinformation would appear more frequently for some topics about GM food, and these topics could have predictive power in predicting misinformation.

Furthermore, propagation related indicators usually consider the characteristics of the propagation tree. Generally, falsehoods could receive much attention, and the false rumors diffused significantly farther and more broadly than the truth [10]. Both reposts and comments can be viewed as responses to the microblogs, which have been used widely in rumor prediction [41, 42]. On Weibo, users express their attitudes through the mark of “like,” which is called attitudes count, and this feature has been proven effective in improving the performance combined with other features [43]. Hence, reposts count, comments count, and attitudes count are used in this study to see if they could contribute to misinformation prediction of GM food.

## Methodology

In this study, we will first compare the three sets of indicators’ prediction performance and then compare the predicting power of the individual indicators within the three sets to explore the mechanism behind the misinformation propagation of GM food. The process includes: (1) propose indicators that could predict false rumors based on domain knowledge, (2) collect data based on keyword filtering, (3) manually label the veracity and sentiment of the messages, (4) analyze topics based on latent Dirichlet allocation (LDA), (5) select indicators for the second round, and (6) compare the performance of the indicators after supervised machine learning through the Microsoft Azure Machine Learning [44] (S1 Fig).

### A. Data collection

255,767 posts ranging from 2009 to 2014 were obtained by filtering the posts of 16 million users of Weibo with the Chinese keyword “转基因,” which is equivalent to a comprehensive set of keywords including genetically modified food, genetically engineered food, gene-edited food, and transgenic food. At the same time, user identities (gender, date of profile creation and date of the message, verification, statuses count, number of friends, number of followees, number of followers) and propagation related indicators (number of attitudes, number of comments, and number of reposts) of the posts were also collected. Then registration age (days between the date of profile creation and date of the message), follow ratio, and diffusion features were calculated based on the collected metadata. The account names of all the users were encoded to preserve user anonymity.

### B. Coding scheme

An important limitation of the misinformation classification task has been the lack of publicly available datasets. Usually, the absence of a reliable dataset for machine learning would significantly affect training and prediction performance. In this work, we initiated a data project from scratch, and the first step was to build up a reliable domain-specific dataset for the following training and prediction.

Among 255,767 posts, the veracity and sentiment of 6,592 posts by 1784 Weibo users were manually coded by eight coders majoring in communication. First, to determine the veracity of GM food posts, coders were trained and guided by a postdoctoral from the field of molecular genetics to gauge the science facts of the posts and used credible websites to figure out the regulations, policies, and others of the posts (S1 Table). The websites are approved as credible sources of information by authorities, and the articles are written by accredited professionals. Second, sentiments determining from the text were coded: positive, neutral, and negative. Posts describing “benefits of GM food, supporting GM food, or evidence that substantiate no harm of GM food” were coded positive; Posts describing “disadvantages and risks of GM food, anti-GMO, infringement of the public’s right to know, or vested interests of pro-GM groups” were coded negative; Other posts were coded neutral. Cohen Kappa scores were calculated to test the intercoder reliability (S2 Table). Finally, to obtain a high-quality dataset for machine learning, we only selected posts that were totally agreed between two coders and removed other posts from the datasets which were not agreed between two coders. So, the intercoder agreement for the final posts obtained for machine learning is 100%. 3,182 posts were agreed between coders and selected as the final dataset for machine learning. The timestamps of 3,182 posts go similarly to that of 255,767 posts from 2009 to 2014 (S2 Fig).

For the measurement of user identities, engagement refers to the users’ seniority and activity level on social media since joining, which could be measured by the total number of statuses, the number of statuses per day, and registration age. The longer a user has been using microblog, the more likely he has developed significant connections with others, and the more likely he would be viewed as a credible user. Reputation can be measured by verification of microblog, number of followers, and follow ratio [45]. Verified users are using authentic identities and verified by Weibo. The follower-followee relationship is asymmetrical. Some users may follow many others to attract them to follow back, and as a result, the number of followers could not accurately predict the actual influence. Follow ratio, scaled ratio of followers over followees, is an alternative measure of reputation. In sum, users verified by Weibo, with a large number of followers, and high follow ratio denote that they enjoy a high reputation. Number of followees, number of followees per day, and number of friends can be used to indicate the extroversion of users on social media. Detailed measurements of the proposed indicators are listed in Table 1.

**Table 1:** Detailed measurements of the proposed indicators.

Variable	Variable	Measure
Credibility	Veracity	Coded as 1 if a rumor is rated as true, and 0 otherwise
**User identity**		
Engagement	Registration age	days between the date of profile creation and date of the message
	Statuses count	The number of posts posted by this user
	Statuses count per day	Statuses count / Registration age
Reputation	Verification	Whether the user is verified by Sina Weibo
	Number of followers	The number of user’s followers
	Follow ratio	The logarithmically scaled ratio of followers over followees: log10 (#followers/#following)
Extroversion on social media	Number of followees	The number of user’s followees
	Number of followees per day	Number of followees/ Registration age
	Number of friends	The number of users who have a mutual following relationship with this user
Gender	Gender	The user’s gender
**Linguistic style**		
Sentiment	Attitude towards GM food	Positive, neutral, and negative
Topic	9 Topics	Topic analysis based on LDA
**Propagation related**		
Propagation	Number of attitudes	The number of attitudes on the post
	Number of comments	The number of comments on the post
	Number of reposts	The number of retweets of the post

### C. Topic modelling

Latent Dirichlet Allocation (LDA) [46], which is a widely used tool for analysis of text data, has been proven effective in detecting deceptive information [40]. LDA is a generative probabilistic model that generates mixtures of latent topics from a collection of documents. Every document is a mixture of topics, and a topic is a probability distribution over words. LDA is a mathematical model, where each document is modeled as a distribution over topics, with topics represented as distributions over words [47].

Before topic modelling, all the texts were preprocessed (S3 Fig). To find the optimal number of topics, we built many LDA models with different values of number of topics and picked the one that gave the highest coherence value [48]. A high score denotes meaningful and interpretable topics. The coherence score obtained increases with the number of topics, with a decline between 9 and 10. However, repeated keywords started to appear in the topic when setting the number of topics as 11. So, the parameter for the number of topics was set as 9 when we built the final LDA model. Table 2 lists the topics obtained through LDA and shows the most frequent terms for each topic.

**Table 2:** Topics of GM food obtained through LDA.

Topic	Most frequent terms in each topic (Chinese translated into English)
Topic 0	Science, doubt, Ministry of Agriculture, harmless, research, scientist, voting, finish, harmful, genetically modified technology, proof, anti-GM food, announcement
Topic 1	Promotion, genetically modified corn, opposition, boycott, Monsanto, development, research, world, academician, Ministry of Agriculture, human, expert, product, import, forwarding
Topic 2	Food, investigation, oil, video, human, science, professor, buy, forbidden, Americans, people, product, bastard, hope
Topic 3	Product, experiment, health, anti-GM food, food, labeling, science, EU, Gu xiulin’s Weibo, principal, method, China Agricultural University, silkworm, products
Topic 4	Cui Yongyuan, illness, eat, discover, suffering, science, McDonald’s, KFC, fries, medical, customer, buy, attitude, expert, tumor
Topic 5	Seed, genetically modified crop, cultivate, farmer, import, science, Ministry of Agriculture, production, control, approval, doubt, grain, genetically modified corn, genetically modified soybean
Topic 6	Oil, special supply, understand, market, search, test, scary, sell, genetically modified food, real, net, things, human body, imagination, network
Topic 7	Test, documentary, child, pig, agriculture, video, feed, investigation, acknowledgment, experiment, turn, expert, genetically modified soybean, go, eat
Topic 8	Food, genetically modified soya bean, import, seed, Hunan, human, approve, Hubei, evidence, end, pesticide, genetic, cultivate, rice, article

### D. Feature selection and model training

**Fig 1:**
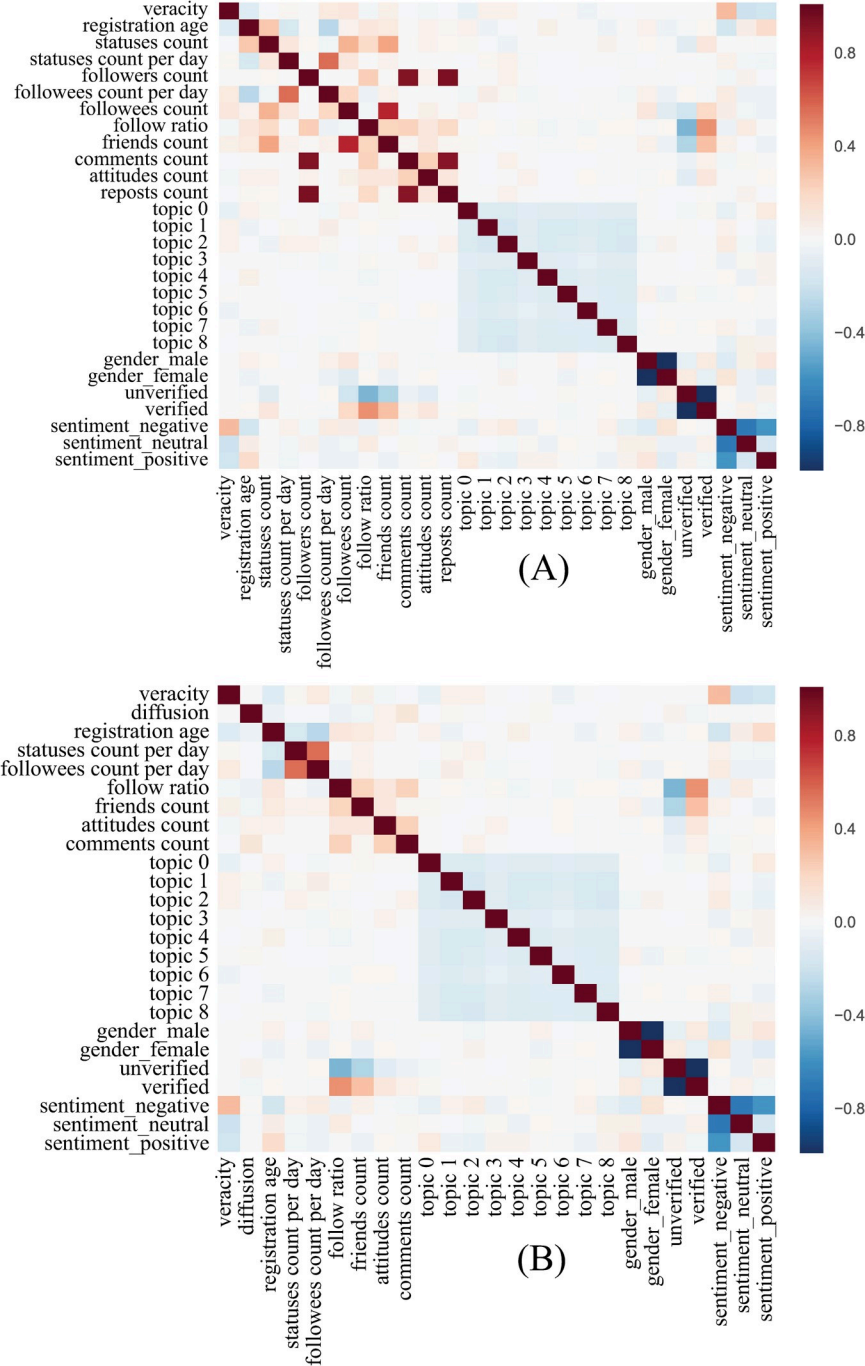
Interdependence between the individual features. (A) before feature engineering; (B) after feature engineering. Red means that features are positively correlated, and blue means that features are negatively correlated.

In machine learning, feature selection is the process of selecting a subset of features that are useful to build a good model, which could help to facilitate data visualization and data understanding, reducing the measurement and storage requirement, and avoiding the curse of dimensionality to improve prediction performance [49]. For the process of feature selection, we followed the checklist of the steps by [49]. First, the features were proposed based on domain knowledge, which includes user identities, linguistic styles, and propagation dynamics. Second, the interdependencies and redundancy were examined, as is shown in Fig 1A. Followers count, reposts count, and comments count are highly intercorrelated. Two of them have to be removed to reduce redundancy, while information from these features should better be preserved. So, a conjunctive feature of diffusion was constructed and added to this set. The conjunctive feature diffusion was created as follows:

Diffusion=(RepostsCount+commentscount)/Followerscount.

As followers count can be deduced from the follow ratio, followees count per day, and registration age, so followers count was removed. Reposts count was also removed, as it can be inferred from other features. Followees count is highly correlated with friends count. Since followees count per day is more representative of extroversion on social media than that of followees count, followees count was not selected. What’s more, statuses count per day can be a better measurement for engagement than that of statuses count, so statuses count was removed. The final selected indicators are shown in Fig 1B.

After the process of feature selection, the final user identities include gender, engagement (measured by registration age and statuses count per day), reputation (measured by verification, number of followers, and follow ratio), and extroversion on social media (measured by number of friends and number of followees per day). The linguistic styles include nine topics and sentiment. The propagation related indicators include diffusion, comments count, and attitudes count.

The experiments were conducted on the Microsoft Azure Machine Learning platform to evaluate all the proposed indicators. First, the data set was split into the training dataset and test set. Then the two-class neural network and two-class logistic regression algorithms respectively were applied to classify GM food posts into misinformation and non-misinformation. Two-class logistic regression is the fast training and linear model, while the two-class neural network is the accuracy and long training time model. The parameters set for the two-class neural network goes as follows:

The number of hidden layers is one.

- The number of nodes in each hidden layer is 100.
- The layers are fully connected.
- The sigmoid activation function is used.
- The initial learning weights diameter is 0.1.
- The learning rate is 0.1.
- The number of learning iterations is 100.

Finally, models were trained and evaluated (S4 Fig). The predicting power of the indicators was analyzed and compared. Over standard statistical models, neural networks have the advantage of discovering patterns that might be hard to be described with simple functions. The neural network model gains its power and develops the ability to learn from training examples. The method of machine learning is not only applied to 20 features and 3,182 observations in this study but also applied to massive datasets in our future study.

## Results

Data analysis was performed to identify the effective indicators of predicting GM food misinformation. It is found that 78.6% of the posts are non-misinformation, and only 21.4% are misinformation, implying that an imbalanced training dataset will be adopted for machine learning (S5 Fig). To avoid the problem of accuracy paradox caused by imbalanced datasets [50], the Receiver Operating Characteristic curve (ROC curve), and the Area Under ROC Curve (AUC score) instead of the accuracy were used to assess the performance of the classifiers in predicting GM food misinformation. The ROC curve is a graphical plot that depicts the trade-offs between the true positive rate (or sensitivity) against the false positive rate (1-specificity) at various threshold settings in the field of machine learning. A random trial will sit on the diagonal with no predictive power, and the ROC curve above the diagonal represents a good result. Similarly, the model would have no discrimination capacity when AUC is 0.5. The higher the AUC, the better the model is at distinguishing between misinformation and non-misinformation.

**Fig 2:**
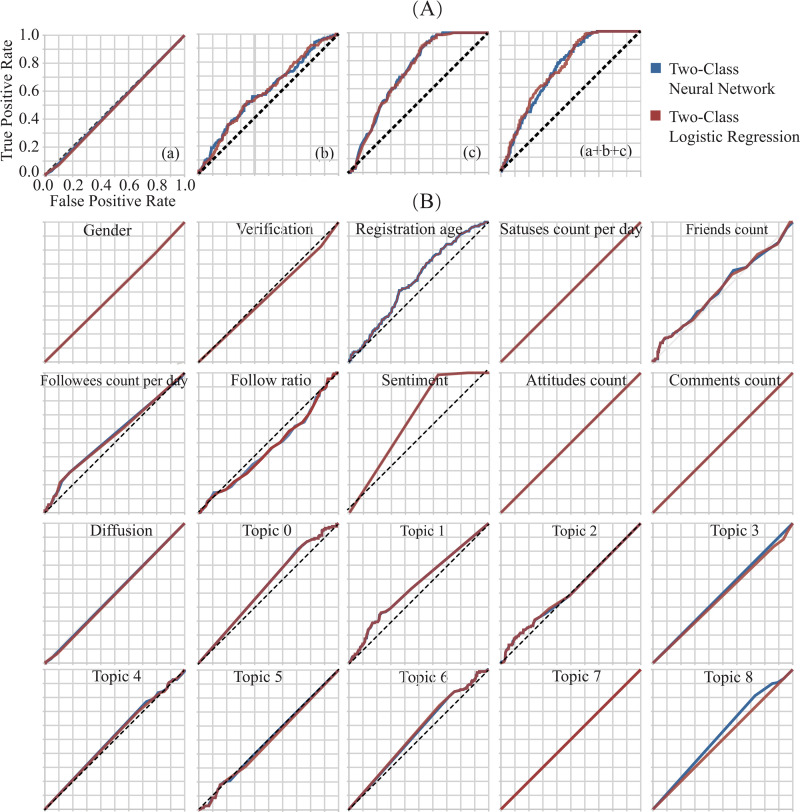
ROC curves for different features. (A) ROC curves for four sets: propagation related indicators (a), user identities (b), linguistic styles (c), and all the indicators (a+b+c). (B) ROC curves of the individual features. The red and blue lines overlap for most of the features.

Fig 2A shows ROC curves for four different sets of indicators. The blue one is generated based on the two-class neural network algorithm, and the red one is generated based on the two-class logistic regression algorithm. The sets are propagation related indicators (a), user identities (b), linguistic styles (c), and all the indicators (a+b+c). Both shapes of the ROC curves and AUC scores generated from the two algorithms are almost the same. The AUC score generated from the two-class neural network for propagation related indicators (a) is 0.494, user identities (b) 0.592, linguistic styles (c) 0.722, and all the indicators (a+b+c) 0.7333. When the AUC of the set of linguistic styles is 0.722, it means there is a 72.2% chance that the model will be able to distinguish between misinformation and non-misinformation. From the shapes of the ROC curves and AUC score, the propagation related indicators do not add any predictive power, user identities have little predictive power, and the linguistic styles outperform the other two sets in predicting misinformation of GM food. When propagation related indicators, user identities, and linguistic styles are combined as input to the classifier, we see that the AUC score leads to an improvement over the individual sets of features. Besides, Accuracy, Precision, Recall, and F1 scores for different sets of features based on the two-class neural network are also provided at the threshold of 0.5 (S3 Table).

To better understand the performance of each individual indicator, ROC curves for individual indicators were explored and plotted. From the ROC curves in Fig 2B, shapes for individual indicators varied within user identities, propagation dynamics, and linguistic styles. Although the overall AUC score of linguistic styles is superior to that of user identities, some of the individual indicators within the set of user identities perform better than those from the set of linguistic styles. Indicators whose AUC score are more than 0.5 are statuses count per day, attitudes count, topic 2, topic 4, friends count, topic 6, topic 0, followees count per day, topic 8, topic 1, registration age, and sentiment, while other indicators whose ROC curves sitting on the diagonal with no predictive power are follow ratio, topic 3, verification, diffusion, gender, topic 5, comments count, followers count, and topic 7.

**Fig 3:**
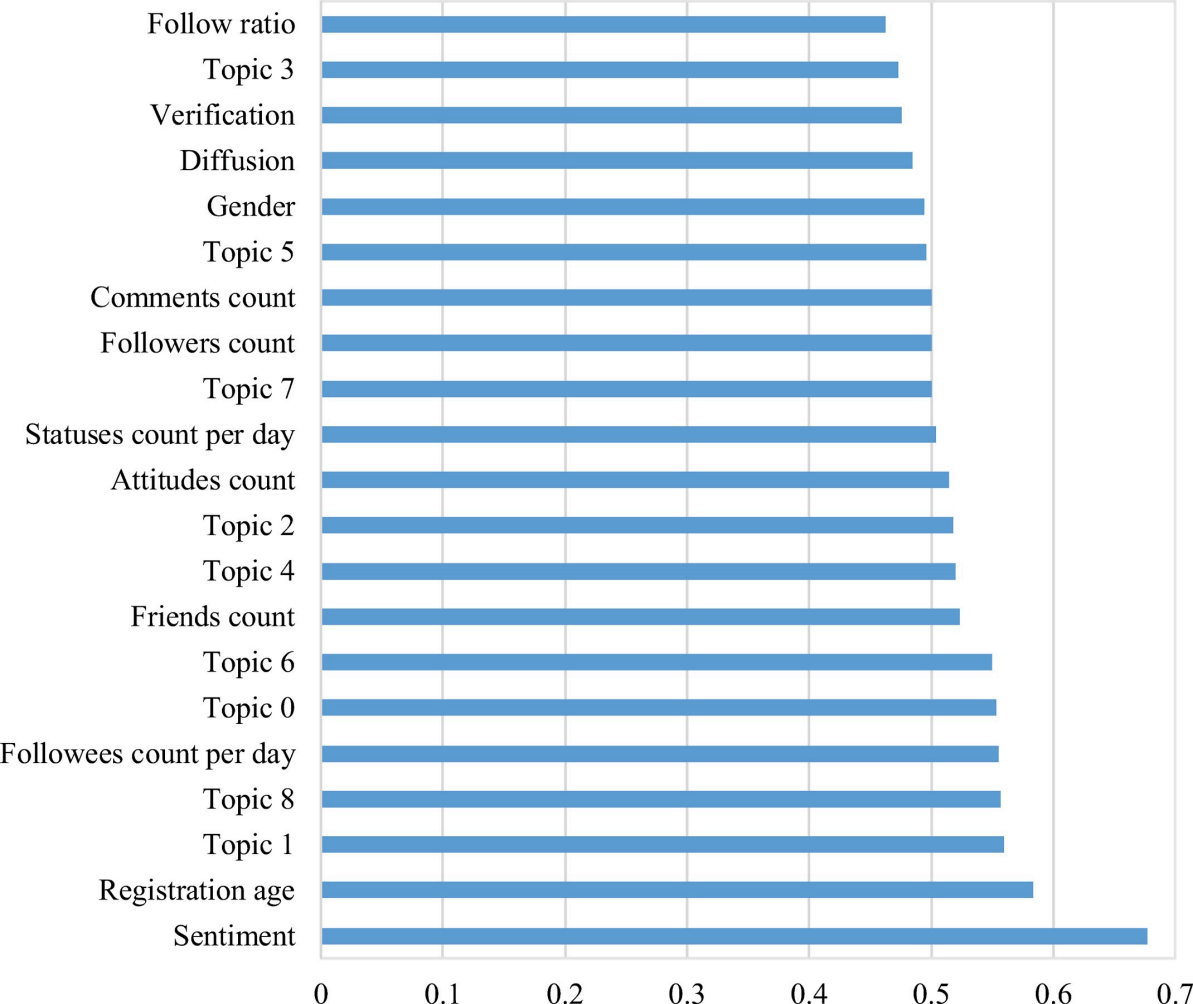
AUC scores of the individual features based on the two-class neural network.

Among the user identities, the AUC scores for engagement (registration age and statuses count per day) and extroversion on social media (followees count per day and friends count) are more than 0.5, while reputation (verification and follow ratio) and gender do not have any predictive power. For linguistic styles, sentiment has the highest AUC score, and different topic has different performance. Fig 3 shows that topic 1, topic 8, topic 0, topic 6, topic 4, and topic 2 have predictive power, whose AUC scores are more than 0.5. The contents of the topics explain their predictive power.

Sentiment outperforms all the other individual indicators, and its AUC score is 0.677, even higher than that of all user identities and propagation related indicators. Most of the posts about GM food are negative, while the numbers of neutral and positive posts are small. For both misinformation and non-misinformation, the negative sentiment has the largest proportion (S6 Fig). There is no positive sentiment for the false rumors, and almost all the sentiments for false rumors are negative, with rather a small amount of them are neutral. The ratio of negative sentiments for misinformation is much higher than that of non-misinformation, indicating sentiment is a good predictor for false rumors of GM food.

## Discussion

As the public increasingly seeks information about science and technology issues online, especially on social media, it is of great importance to present the public with scientific information. However, with the explosion of content on social media, it is hard for journalists, policymakers, and citizens to evaluate the credibility of the content. And addressing the problem of online misinformation should be a top priority. Identifying the indicators of false rumors will help people determine the microblogs’ credibility, which will enhance a scientific understanding of GM food and provoke appropriate public discussion.

Detection of GM food misinformation is different from that of general types of misinformation. Technologies related to GM food are usually cutting-edge. Consequently, science communication about this technology is insufficient, and many people are not engaged in the topic. The public has very limited knowledge of genetic engineering. Their concerns regarding this issue are not only limited to health and environment, but also ethics, corporate monopolies, and the right to know, which would trigger negative sentiments such as fears and anxiety. Hence, people may spread GM food rumors to relieve the tension of anxiety, since they may seek information, authenticate their comments with suitable references, and express (at time tentatively) anxiety, belief, and disbelief [51]. Negative sentiment was strong among the public when talking GM food, which explains why the linguistic styles have strong predictive power for misinformation of GM food.

Moreover, the set of linguistic styles (topics and sentiment) is better than those of user identities and propagation-related indicators in detecting GM food’s false rumors. Within the linguistic styles, sentiment has the best predictive power, and the set of topic-based features do not perform as well as the sentiment. This study revealed that some indicators employed in general rumor and misinformation detection do not have predictive power in GM food false rumor detection, indicating that some indicators are specific to an event.

### A. Sentiment is the strongest indicator for predicting the misinformation of GM food

Performance result using sentiment is significantly better than those using other individual indicators in misinformation detection of GM food. In this study, posts are classified as expressing positive, neutral, and negative opinions. For misinformation, almost all the posts are negative, and there are no positive posts, indicating that negative users are very active and aggressive. However, non-misinformation is highly associated with neutral and positive sentiments, which may be a special case and only applied to the issue of GM food.

Generally, posts about science education or scientific introduction to GM technology were labeled neutral or positive. The dominant sentiment of the posts is negative, and the number of positive and neutral posts is much less than that of negative posts, indicating there is a scarcity of contents of popular science about this technology on Weibo. According to the interdependence of the indicators, the negative sentiment is more associated with topic 1, topic 2, and topic 4, which have better predictive power than other topics. These three topics are about opposition to the technology and public debate after Cui Yongyuan’s documentary investigating US people’s attitude toward GM food and its controversy in the US. Public debate has extended from the basic concept of safety to other political issues, like conspiracy orchestrated by Western countries or interest groups, which easily triggered negative sentiment.

One possible interpretation for why sentiment is a far-above-chance predictor for misinformation is that when people feel negative towards GM food, they may suffer from anxiety, uncertainty, fear, and doubt. In the absence of information from authorities explaining ambiguity and uncertainty, members within a community may engage in a collective problem-solving process: share and evaluate information to eliminate their uncertainty [26]. Hence, when people need to acquire an understanding or knowledge of GM food, they will post rumors to relieve uncertainty.

### B. Misinformation of GM food is more likely to be present in risk-related topics

Topics that talk about risks and ethics are more associated with GM related misinformation. According to Fig 3, risk-related topics, such as Topic 0, 6, and 8, significantly outperform topics related to policy and economy (e.g., Topic 3 and 5) in terms of the AUC score or prediction performance. Topic 0 discussed whether GM food is safe, Topic 6 describes that some people believe GM technology is a form of bioterrorism targeted at specific groups and non-GM oil is provided for high-status people, and Topic 8 is about illegal GM rice being grown in Hubei province. Topic 3 concerns labeling policy, and Topic 5 the import of GM food. Besides, Topic 2 and 4 are about Cui Yongyuan, a famous host from CCTV, who filmed a documentary about GM food in the US at the end of 2013, which sparked heated discussion on Weibo. And Topic 7 relates to the golden rice experiment conducted on children.

The better performance of risk-related topics could be explained in two aspects. One is when people are uncertain about the risks of GM technology, they may post false rumors as a way of fact-finding. Online users may post their concerns about the risks, which could be rumors, to reduce the uncertainty that coheres with the idea that the dominant psychological motivation of spreading rumors: uncertainty reduction and sense-making [52]. Another possibility of why risks and ethics are associated with false rumors could be that risk-related topics are very eye-catching. One of the top motivations of spreading rumors is enhancing, building, and maintaining a relationship [26]. People spread false rumors about risks in order to interact with others and draw more attention.

### C. Reputation has almost no predictive power for misinformation of GM food

This study shows that reputation measured by verification and follow ratio has almost no predictive power, indicating users of high reputation could also spread false rumors about GM food on Weibo. On the contrary, for the credibility assessment of general types of misinformation, the previous study shows that high-reputation users with many followers are inclined to spread more credible information [18]. Users with a high follow ratio or being verified enjoy high reputation and prestige on social media. These users are very cautious about posting messages on social media, as they fear that spreading rumors, especially false rumors, will harm their reputation, and it would take much effort to repair and restore their reputation. While in the case of GM food, high reputation users on Weibo spread false rumors as often as low reputation users. It may imply that some high reputation users have a strong standpoint but limited knowledge of GM food.

Several explanations may justify this phenomenon. First, if high reputation users are not equipped with relevant knowledge about genetic engineering, they would not be able to distinguish misinformation from non-misinformation and may spread rumors about GM food. Second, processing and sharing behavior is a way of strengthening reputation in one’s social network [26]. In times of uncertainty, information about GM food is even more valuable. High reputation users would demonstrate their status of being “in the know” and unwittingly spread false rumors to gain respect and liking from other people.

### Implications for science communication

This current study provides important theoretical and practical implications and contributions to our understanding of credibility evaluation for GM food information on social media. From the theoretical perspective, we extended prior research by exploring credibility indicators to predict GM food misinformation. The effective factors for the special case GM food misinformation are different from general types of information. For example, general types of tweets, including negative sentiment, are associated with credible content [18], while GM food misinformation is more likely to be related to negative sentiment. Reputation has almost no predictive power for misinformation of GM food, which is different from previous research. Therefore, the effectiveness of indicators may vary in different contexts. Taking the context into consideration would help researchers distinguish falsehood efficiently. In addition, the risk-related topic is an effective predictor for GM food misinformation. Researchers could consider categorizing the topics into risk and non-risk when extracting topic features in credibility research.

From a practical perspective, this study provides readers a strategy for assessing the credibility of messages on social media based on credibility indicators. Online readers can evaluate the credibility of GM food posts based on the message and the users’ metadata. Check the sentiment and the sensitive topics of the posts. Most of the false rumors are negative and play on readers’ anxiety and uncertainty about GM technology; in addition, misinformation tends to center on risk-related topics. Readers should be careful about the credibility of microblogs with extreme sentiment; Also, online readers have to pay attention to the posts if the posts are from users who are extraverts on Weibo or from users whose engagement is very low on Weibo. In the case of GM food, high reputation users on Weibo spread false rumors as often as low reputation users.

What’s more, this study offers social media platforms a way of debunking misinformation to enhance science communication and contribute to the policy-making process of GM technology. The Weibo platform is supposed to display better and high-quality searching results to better convey credibility. Only manual review and labeling of post credibility is increasingly difficult in the Internet age. The platform should use effective indicators to automatically identify the misinformation about GM food from the massive amount of content quickly and efficiently and provide warnings of credibility to the public. Although users who enjoy a high reputation on social media are inclined to spread more credible information, they are not equipped with knowledge of genetics and could also spread misinformation about GM food. Among high reputation users, only professionals specialized in genetic engineering are credible sources. Hence, in order to combat misinformation, Weibo might consider amplifying the voices of the professions to make credible information more accessible and influential.

### Limitations

However, several limitations should be noted. First, we coded the 6,592 GM related posts from 2009 to 2014 by 1,784 Weibo users for their veracity. The 1,784 users are mainly from Shanghai. Future work will be extended to more areas in China or even the world. Second, we were unable to categorize the sentiment into detailed categories. More efforts are needed to rate the extent of negativity for both the misinformation and non-misinformation. In the future, the degree of polarity on a specific opinion should be more precise and mapped onto more detailed categories: very positive, positive, neutral, negative, and very negative, or onto numerical sentiment score on a scale of -1 (very negative) to +1 (very positive). The more refined classification of sentiment will help to improve the accuracy of misinformation prediction.

## Supporting information

S1 Fig The framework of the method. (DOCX)

S2 Fig (A) Timestamps of 255,767 GMO posts; (B) timestamps of 3,182 GMO posts.
(DOCX)

S3 Fig Flowchart of preprocessing the Chinese post before topic modeling. (DOCX)

S4 Fig Flowchart of supervised machine learning. (DOCX)

S5 Fig Distribution of proposed indicators. (DOCX)

S6 Fig (A) Sentiment distribution among all the posts; (B) sentiment distribution among misinformation and non-misinformation.
(DOCX)

S1 Table Codebook for common misinformation on Sina Weibo. (DOCX)

S2 Table The Cohen’s Kappa scores were calculated for labeling tasks of the coders. (DOCX)

S3 Table AUC, Accuracy, Precision, Recall, and F1 scores for different sets of features based on two-class neural network. (DOCX)
