# Supplementary figures and images for: Microblog credibility indicators regarding misinformation of genetically modified food on Weibo
Source: PLoS One. 2021 Jun 1;16(6):e0252392. doi: 10.1371/journal.pone.0252392 (PMC8168881; doi:10.1371/journal.pone.0252392)

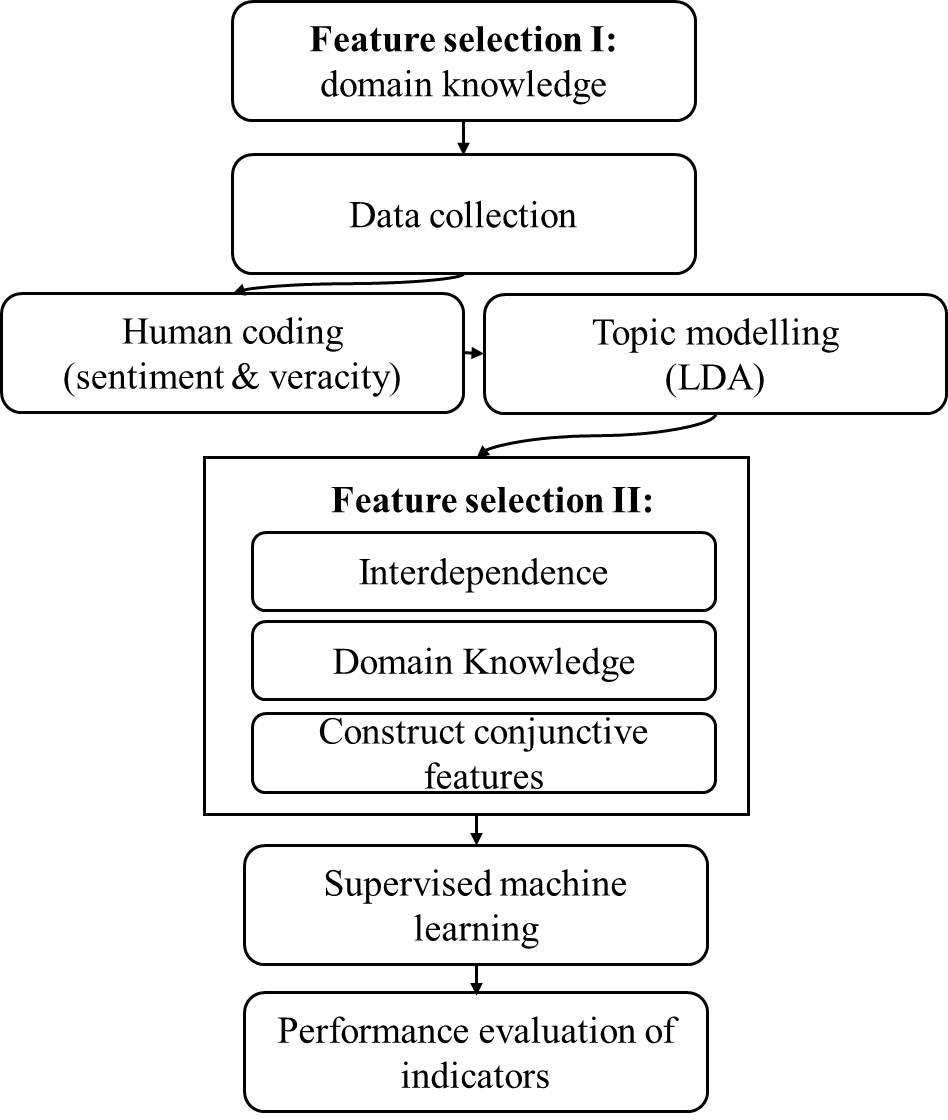


**S1 Fig. The framework of the method.**

Supplement: S1 Fig — (DOCX) [file pone.0252392.s001.docx]

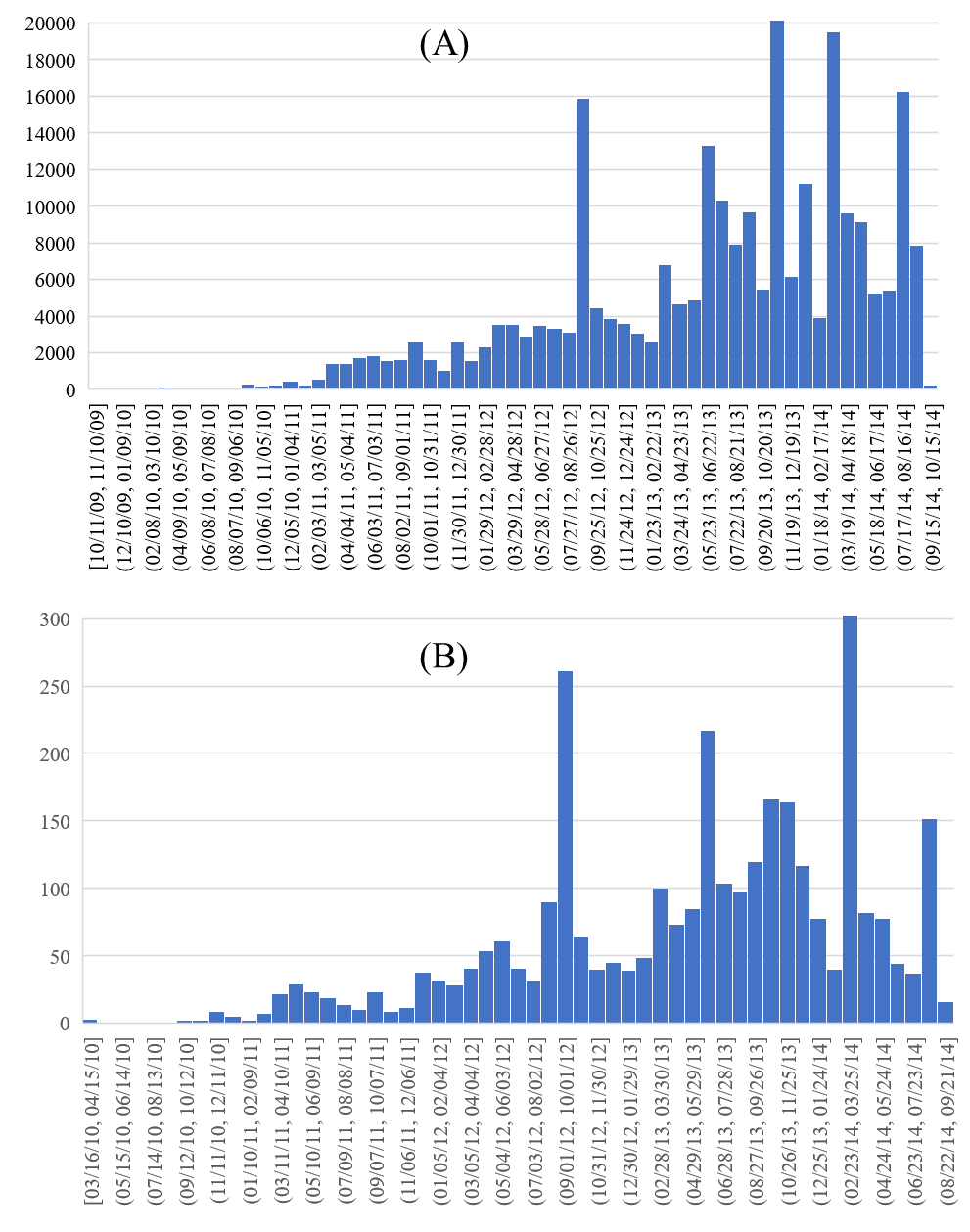


**S2 Fig. (A) Timestamps of 255,767 GMO posts; (B) timestamps of 3,182 GMO posts.**

Supplement: S2 Fig — (A) Timestamps of 255,767 GMO posts; (B) timestamps of 3,182 GMO posts. (DOCX) [file pone.0252392.s002.docx]

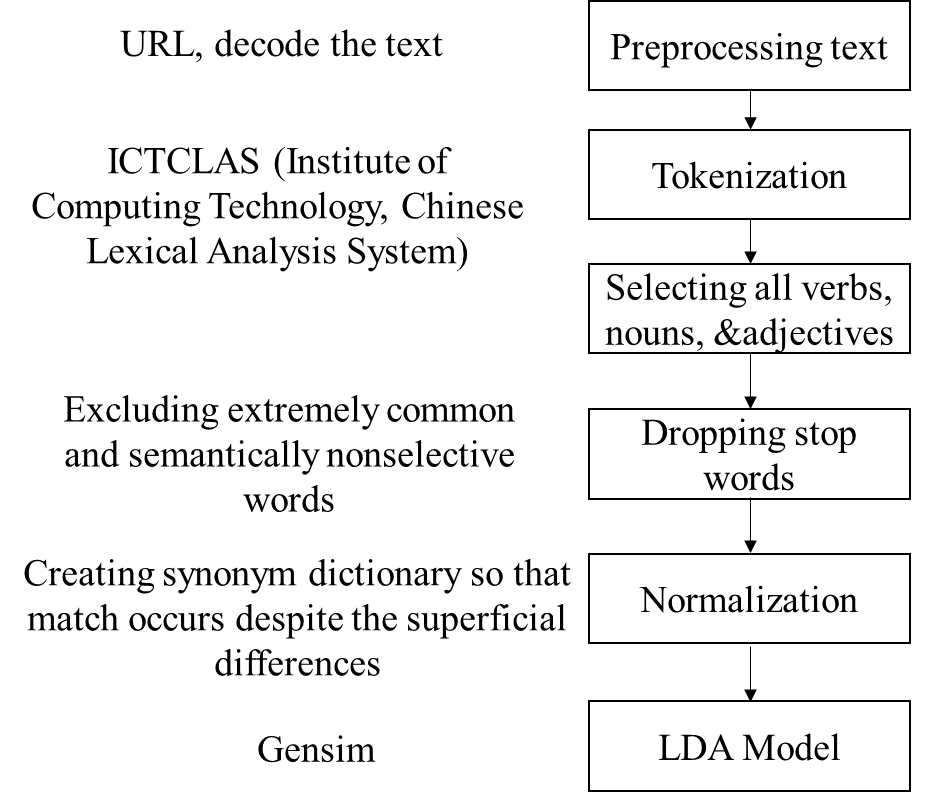


**S3 Fig. Flowchart of preprocessing the Chinese post before topic modeling.**

Supplement: S3 Fig — (DOCX) [file pone.0252392.s003.docx]

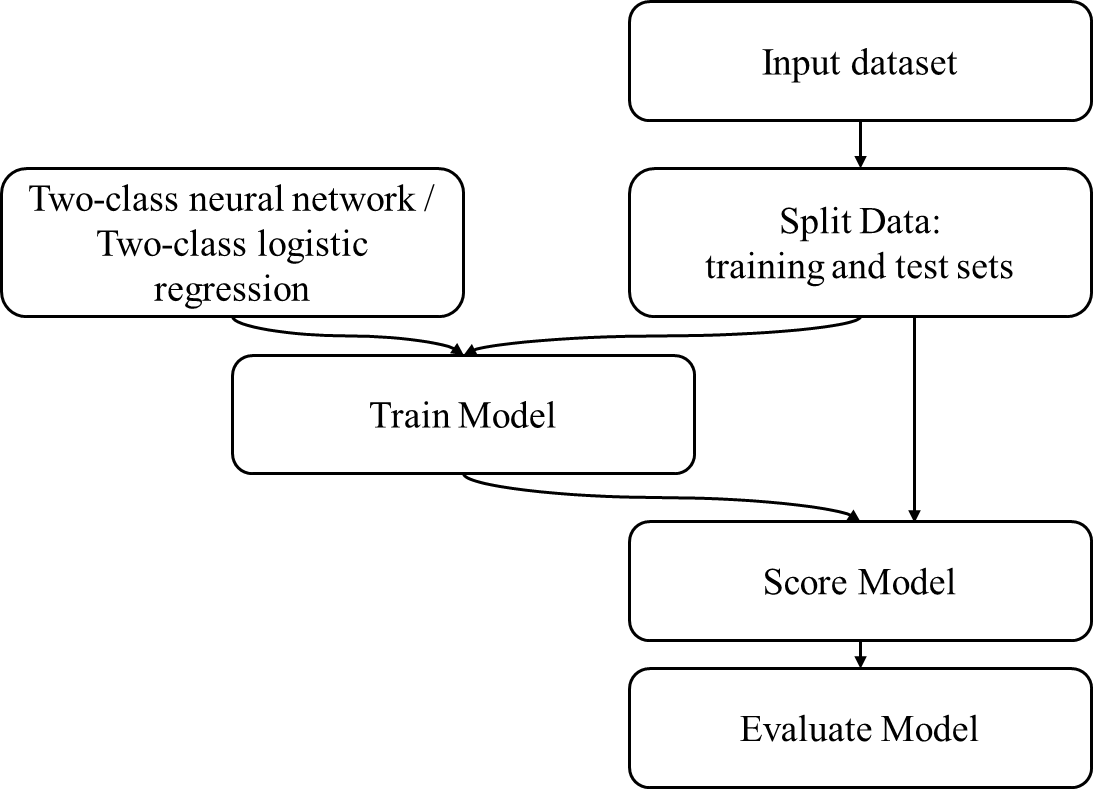


**S4 Fig. Flowchart of supervised machine learning.**

Supplement: S4 Fig — (DOCX) [file pone.0252392.s004.docx]

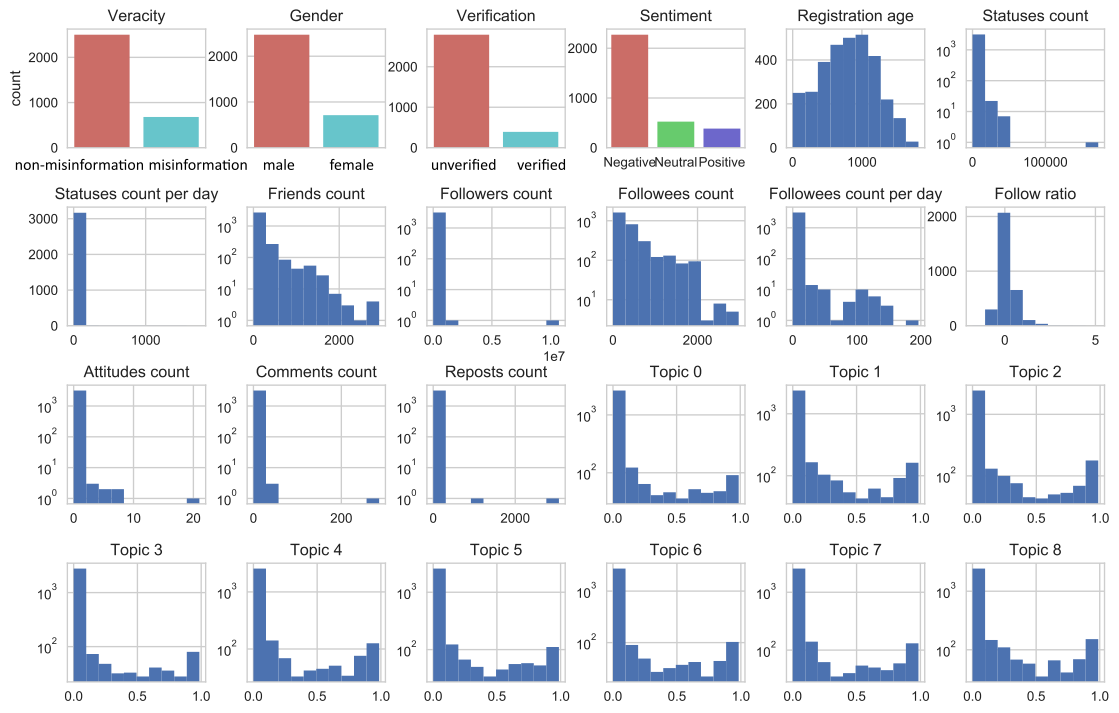


**S5 Fig. Distribution of proposed indicators.**

Supplement: S5 Fig — (DOCX) [file pone.0252392.s005.docx]
