# Supplementary material for: Microblog credibility indicators regarding misinformation of genetically modified food on Weibo
Source: PLoS One. 2021 Jun 1;16(6):e0252392. doi: 10.1371/journal.pone.0252392 (PMC8168881; doi:10.1371/journal.pone.0252392)
Supplement: S6 Fig — (A) Sentiment distribution among all the posts; (B) sentiment distribution among misinformation and non-misinformation. (DOCX) [file pone.0252392.s006.docx]

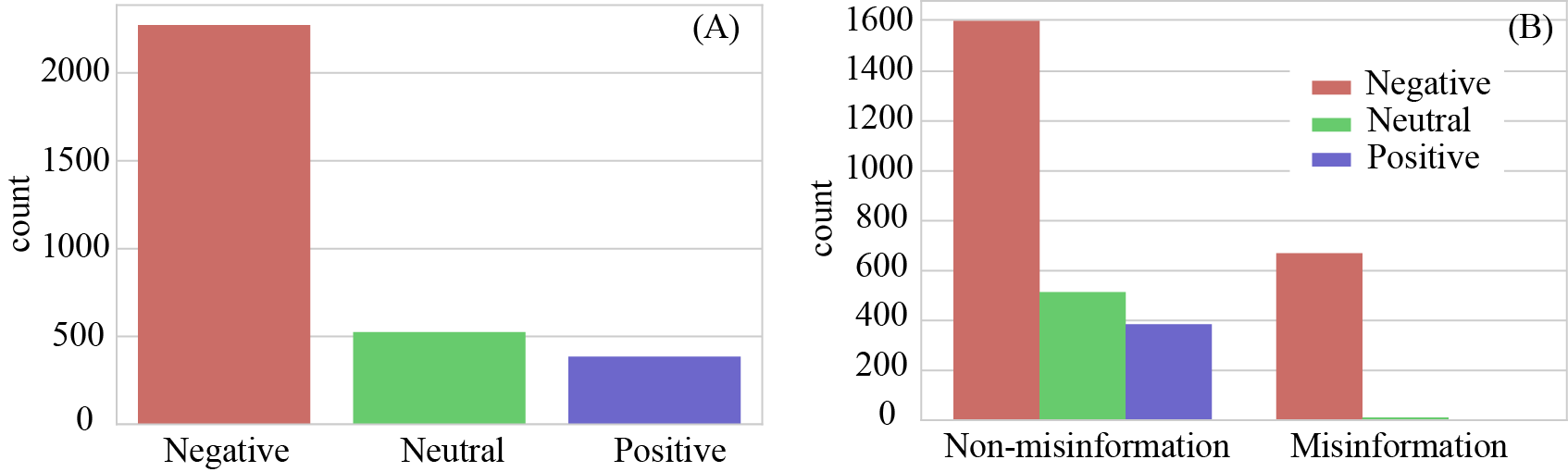


**S6 Fig. (A) Sentiment distribution among all the posts; (B) sentiment distribution among misinformation and non-misinformation.**
