# Supplementary material for: Microblog credibility indicators regarding misinformation of genetically modified food on Weibo
Source: PLoS One. 2021 Jun 1;16(6):e0252392. doi: 10.1371/journal.pone.0252392 (PMC8168881; doi:10.1371/journal.pone.0252392)
Supplement: S1 Table — (DOCX) [file pone.0252392.s007.docx]

**S1 Table. Codebook for common misinformation on Weibo.**

| **Misinformation on Sina Weibo** | **URLs of proof** | **Facts** |
| --- | --- | --- |
| GM food (such as GM corn) will cause infertility, lower the fertility, and affect offspring. | http://sitn.hms.harvard.edu/flash/2015/will-gmos-hurt-my-body/ | GM food (such as GM corn) will not cause infertility, lower the fertility, or affect offspring from the research of independent groups worldwide. |
| Genetically modified maize (corn) induces tumors in rats and humans. | https://allianceforscience.cornell.edu/blog/2018/06/european-studies-disprove-seralinis-gmo-maize-tumor-claims/ | Three European studies have disproved genetically modified maize (corn) induces tumors in rats and humans. |
| Identify GM produce by their appearances such as size, shape, and color. | http://shipin.people.com.cn/n/2013/1128/c85914-23678869.html | GM produce cannot be identified by their appearances such as size, shape, and color. |
| GMOs will change our genes or the ability of the modified DNA to transfer to the DNA of whomever eats it or have other toxic side effects. | http://sitn.hms.harvard.edu/flash/2015/will-gmos-hurt-my-body/ | GMOs will not change our genes or the ability of the modified DNA to transfer to the DNA of whomever eats it or have other toxic side effects. |
| The tiny size of cherry tomatoes comes from the genetically modification. | https://en.wikipedia.org/wiki/Cherry_tomato | The tiny size of cherry tomatoes does not come from the genetically modification. |
| The deep-purple coloring of purple sweet potato comes from the genetically modification. | https://www.sciencedaily.com/releases/2009/06/090629132250.htm | The deep-purple coloring of purple sweet potato does not come from the genetically modification. |
| The color of different color bell pepper are genetically modified. | https://en.wikipedia.org/wiki/Bell_pepper#cite_note-8 | The color of different color bell pepper are not genetically modified. |
| Cherry tomatoes, purple sweet potatoes, and color bell pepper sold on Chinese market are genetically modified. | http://www.moa.gov.cn/ztzl/zjyqwgz/sjzx/201708/t20170803_5768417.htm | Currently, cherry tomatoes, purple sweet potatoes, or color bell pepper sold on Chinese market are not genetically modified. |
| China has given any safety approval to commercial production of genetically modified grains. | http://politics.people.com.cn/n/2015/0306/c70731-26651467.html | China has spent billions on research of biotech crops, but it has not yet approved the planting of any GM varieties of staple food crops. |
| The genetically modified soybean products are illegally sold on the market. | http://www.agrogene.cn:8081/info-3743.shtml | The genetically modified soybean products are legally sold on the market. |
| China has not established GM food labeling regime. | https://www.loc.gov/law/help/restrictions-on-gmos/china.php | GMO products on the GMO list published by the state must be clearly labeled when sold within China; unlabeled products may not be sold. |
| People in the United States do not eat GM food | https://onlinelibrary.wiley.com/doi/full/10.1111/j.1539-6924.2005.00668.x | A lot of processed foods containing ingredients from engineered canola, soybeans and corn have been sold in the US supermarkets for a long time |
| Consumers can tell that the item is genetically modified by its bar code (the five-digit code would start with an 8 if it was genetically modified) | https://ifpsglobal.com/Portals/22/IFPS%20Documents/PLU%20FAQ/PLU%20Site%20FAQs%20Aug%202015%20v2.pdf | Though the '8' prefix (83000-84999) was once reserved for GMO produce items, the prefix was never used at retail. |
