# Supplementary material for: Microblog credibility indicators regarding misinformation of genetically modified food on Weibo
Source: PLoS One. 2021 Jun 1;16(6):e0252392. doi: 10.1371/journal.pone.0252392 (PMC8168881; doi:10.1371/journal.pone.0252392)
Supplement: S2 Table — (DOCX) [file pone.0252392.s008.docx]

**S2 Table. The Cohen's Kappa scores were calculated for labeling tasks of the coders.**

|  | Rater1&rater2 | Rater3&rater4 | Rater5&rater6 | Rater7&rater8 |
| --- | --- | --- | --- | --- |
| Sentiment | 0.560 | 0.557 | 0.602 | 0.651 |
| Veracity | 0.525 | 0.506 | 0.576 | 0.586 |

*Note*: The Kappa score can be interpreted as follows: <0.2 regarded as poor, 0.21-0.40 as fair, 0.41-0.60 as moderate, 0.61-0.80 as good, and >0.8 as very good (Altman, 1990). Some ambiguous posts which were not agreed on by two coders could influence the performance of machine learning. The ambiguous posts were removed from the training dataset directly.

To obtain a high-quality dataset for machine learning, we only selected posts that were totally agreed by two coders and removed other posts from the datasets which were not agreed by two coders. So the intercoder agreement for the final posts obtained for machine learning is 100%.
