# Supplementary material for: Microblog credibility indicators regarding misinformation of genetically modified food on Weibo
Source: PLoS One. 2021 Jun 1;16(6):e0252392. doi: 10.1371/journal.pone.0252392 (PMC8168881; doi:10.1371/journal.pone.0252392)
Supplement: S3 Table — (DOCX) [file pone.0252392.s009.docx]

**S3 Table. AUC, Accuracy, Precision, Recall, and F1 scores for different sets of features based on two-class neural network.**

| Features | AUC | Accuracy | Precision | | Recall | | F1 Score | True Positive | False Negative | False Positive | True Negative |
| --- | --- | --- | --- | --- | --- | --- | --- | --- | --- | --- | --- |
| propagation related indicators (A) | 0.494 | 0.781 | 1.000 | 0.000 | | 0.000 | | 0 | 139 | 0 | 497 |
| User identities (B) | 0.592 | 0.783 | 0.667 | 0.014 | | 0.028 | | 2 | 137 | 1 | 496 |
| Linguistic styles (C) | 0.722 | 0.781 | 1.000 | 0.000 | | 0.000 | | 0 | 139 | 0 | 497 |
| All features (A+B+C) | 0.733 | 0.777 | 0.333 | 0.022 | | 0.041 | | 3 | 136 | 6 | 491 |
